# Recovery and characterization of a *Citrus clementina *Hort. ex Tan. 'Clemenules' haploid plant selected to establish the reference whole Citrus genome sequence

**DOI:** 10.1186/1471-2229-9-110

**Published:** 2009-08-22

**Authors:** Pablo Aleza, José Juárez, María Hernández, José A Pina, Patrick Ollitrault, Luis Navarro

**Affiliations:** 1 Centro de Protección Vegetal y Biotecnología, Instituto Valenciano de Investigaciones Agrarias (IVIA), Ctra. Moncada-Náquera km 4.5, 46113 Moncada, Valencia, Spain; 2 Unité de Recherche Multiplication Végétative, Centre de Coopération Internationale en Recherche Agronomique pour le Développement (CIRAD), Montpellier 34398, France

## Abstract

**Background:**

In recent years, the development of structural genomics has generated a growing interest in obtaining haploid plants. The use of homozygous lines presents a significant advantage for the accomplishment of sequencing projects. Commercial citrus species are characterized by high heterozygosity, making it difficult to assemble large genome sequences. Thus, the International Citrus Genomic Consortium (ICGC) decided to establish a reference whole citrus genome sequence from a homozygous plant. Due to the existence of important molecular resources and previous success in obtaining haploid clementine plants, haploid clementine was selected as the target for the implementation of the reference whole genome citrus sequence.

**Results:**

To obtain haploid clementine lines we used the technique of *in situ *gynogenesis induced by irradiated pollen. Flow cytometry, chromosome counts and SSR marker (Simple Sequence Repeats) analysis facilitated the identification of six different haploid lines (2*n *= *x *= 9), one aneuploid line (2*n *= 2*x*+4 = 22) and one doubled haploid plant (2*n *= 2*x *= 18) of 'Clemenules' clementine. One of the haploids, obtained directly from an original haploid embryo, grew vigorously and produced flowers after four years. This is the first haploid plant of clementine that has bloomed and we have, for the first time, characterized the histology of haploid and diploid flowers of clementine. Additionally a double haploid plant was obtained spontaneously from this haploid line.

**Conclusion:**

The first haploid plant of 'Clemenules' clementine produced directly by germination of a haploid embryo, which grew vigorously and produced flowers, has been obtained in this work. This haploid line has been selected and it is being used by the ICGC to establish the reference sequence of the nuclear genome of citrus.

## Background

In recent years, the development of structural genomics has generated a growing interest in obtaining haploid plants. The recovery of haploid and double haploid plants from gametic embryogenesis enables homozygous lines to be isolated in a single step, whereas only near-homozygous genotypes can be obtained through several generations of selfing in classical genetic approaches [1]. Moreover, such traditional methods are extremely difficult to implement in woody species, such as citrus, which are highly heterozygotic and have long juvenile phases, requiring several decades to obtain a near-homozygous plant.

Haploid and double haploid lines play an important role in genomics [2,3] and have been used for physical mapping [4], genetic mapping [5-7] and for the integration of genetic and physical maps [8], thereby permitting high precision analyses of the relationship between megabases and centimorgans and, thus, increasing the precision in labelling candidate genes [9,10]. Additionally, haploid and double haploid plants are adapted for mutagenesis and genetic transformation experiments, presenting the advantage of immediate production of homozygous lines [11]. It is expected that, in the near future, haploid and double haploid plants will play an increasingly important role in whole genome sequencing (WGS) projects, where homozygosity is a particular advantage. The WGS from genotypes with high levels of heterozygosity generate problems in alignment between physical and linkage maps due to an incorrect order of the BAC (Bacterial Artificial Chromosome) clones within a contig producing apparent duplication of *loci *in the physical map, the assembly of BAC clones corresponding to the two different haplotypes into separate contigs [12] and the difficulty to distinguish alleles at the same *locus *from paralogs at different *loci *in two divergent haplotypes [13]. Polymorphism in a whole genome sequence complicate the assembly process, display lower quality and assembly contiguity and completeness is significantly lower than would have been expected in the absence of heterozygosity [13]. For instance, the WGS of grapevine was made from a near-homozygous line obtained after six successive self-pollinated generations [14,15].

Commercial citrus varieties are characterized by high heterozygosity [16]. The recent comparison of blind versus "known-haplotype" assemblies of shotgun sequences obtained from a set of BAC clones from the heterozygous sweet orange [17] led the ICGC to the decision in 2007 to establish the reference sequence of the Citrus genome from an homozygous genotype.

Considering the long juvenile period, and the very frequent presence of self-incompatibility in citrus, thereby making it almost impossible to obtain near-homozygous plants by succesive selfing steps, it was decided to use a haploid plant for sequencing. The clementine (*C. clementina *Hort. ex Tan.) was chosen as the reference species for the *Citrus *genus because: a large number of SSR is available [18,19], ESTs (Expressed Sequence Tag) [20] and microarrays have been developed for functional analysis [21], BAC libraries have been characterized in the perspective of physical mapping [22], genetic maps are under development [23,24] and it has already been proved possible in the past to obtain haploid clementine lines [[25,26], the present paper]. Moreover, clementines are the main group of cultivars for mandarin fresh-fruit market and constitute an essential germplasm for mandarin breeding. Clementine is a natural hybrid between sweet orange and common mandarin selected in 1902 in Algeria. All the cultivars of clementine have arisen from the initial 'Fina'clementine by the accumulation of spontaneous mutations. Among them, 'Clemenules' clementine, a direct mutation of 'Fina', is the most commercially important cultivar in the Mediterranean Basin and has been selected as target to obtain the haploid genotype for whole genome sequencing.

Androgenesis has been the most commonly employed approach to obtain haploid, aneuploid, double haploid and trihaploid plants in citrus [27-33]. Generally attainment of haploid, double haploid and trihaploid plants using this methodology requires complex culture media with several growth regulators, formation of calli and, in all cases, long culture periods. Due to the regeneration methods, a higher incidence of somaclonal variation should be expected in plants derived from male cells [34]; moreover, the callus stage has generally been proved to generate somaclonal variants in citrus [35,36].

Gynogenesis is an alternative technique for producing haploid plants. It has been successfully applied in fruit trees such as *Actinida deliciosa *[37], *Malus domestica *(L.) Borkh [38] and *Pyrus comunis *(L.) [39]. In cherry tree, *Prunus *spp. [40] and kiwi, *Actinidia deliciosa*, [37] double haploid plants have been obtained by spontaneous gynogenesis.

Gynogenesis also occurs in citrus. It has been observed in hybridizations 2*x *× 2*x *and 2*x *× 3*x *[41-43]. Germanà and Chiancone [44] obtained haploid clementine by pollinating *in vitro *pistils of clementine with pollen of the triploid hybrid 'Oroblanco' (*C. grandis *× *C. paradisi*). Gynogenesis induced by irradiated pollen is another technique that can be used to obtain haploid plants. Haploid embryogenic calli and haploid plants have been obtained after pollination of clementine flowers with irradiated pollen of 'Meyer' lemon (*C. meyeri *Y. Tan.) and embryo rescue [25]. Later, using the same technique, Froelicher *et al*. [45] obtained haploid plants of clementine, 'Fortune' mandarin (*C. tangerina *× *C. clementina*) and 'Ellendale' tangor (*C. reticulata *× *C. sinensis*). Gamma ray doses between 150 and 900 Grays effectively generated haploid plants in these experiments. Nevertheless, the generation of haploid plants with this technique is not easy and is generally inefficient, with very few plants becoming established in the greenhouse.

Most of the reports on citrus haploid plants mention the very low vigour of these genotypes and a lot of them died after a few months of culture in culture tubes or greenhouse [25,31,40,44,45]. One of the requirements of the ICGC for the whole genome sequencing project was to select a homozygous plant with vigorous growth.

In this paper we describe the recovery of haploid, aneuploid and double haploid plants of 'Clemenules' clementine by gynogenesis *in situ*, induced by irradiated pollen of 'Fortune' mandarin. Cytogenetic and SSR analysis facilitated determination of the origin of these different genotypes. Additional morphological and histological studies, in comparison with the parental diploid 'Clemenules' clementine, were conducted for one haploid line with vigorous growth and easily extractable DNA. This plant has been selected by the ICGC to establish the reference sequence of the whole nuclear genome of citrus, which has been launched early in 2009.

## Results

### Recovery of plants and ploidy level analysis

**Figure 1:**
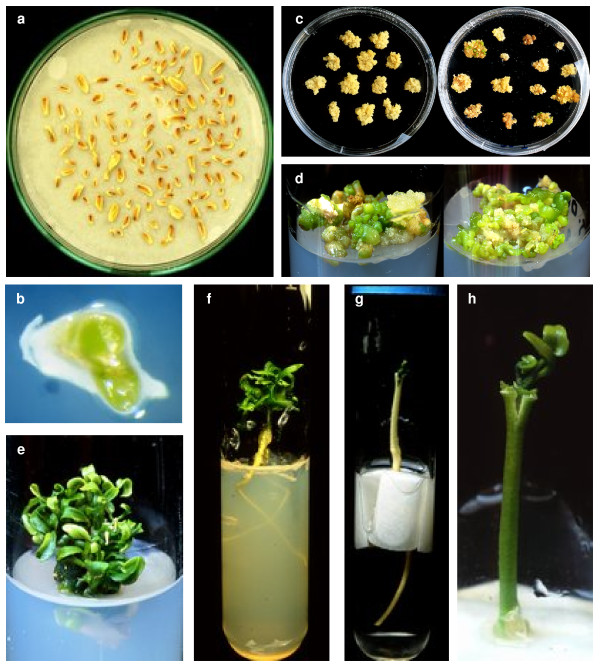
***a*. Small seeds of 'Clemenules' obtained from pollination with irradiated pollen**. *b*. Embryo present in seeds. *c*. Embryogenic calli originating from embryo culture. *d*. Cluster of embryos obtained from embryogenic calli. *e*. Shoots produced by embryos regenerated from embryogenic calli. *f*. Regenerated plant from direct germination of embryo without a callus phase. *g*, *h*. *In vitro *micrograft of haploid shoot.

After pollination of 350 'Clemenules' clementine flowers with irradiated pollen of 'Fortune'mandarin, we obtained 270 fruits containing 1744 seeds approximately 4-5 mm in length, much smaller than normal seeds (10-12 mm on average). Only 2.9% of these seeds contained embryos (Figures 1a and 1b). A total of 51 embryos were cultivated *in vitro*, 13 of which developed either by direct germination or through the formation of embryogenic calli (Figures 1c, d, e and 1f). To regenerate plants it was necessary to use the technique of shoot tip grafting *in vitro *[46] because embryos did not develop roots and when they produced roots they were small and very weak. Nine plants were obtained by direct germination of embryos and subsequent *in vitro *micrografting (Figures 1g and 1h). Four embryogenic calli were also induced (Table 1) producing a total of 96 embryos, from which 16 plants were recovered by *in vitro *micrografting of resulting shoots.

**Table 1:** Ploidy level of embryogenic calli and somatic embryos obtained.

Callus	Ploidy	N° obtained embryos	N° germinated embryos	N° obtained plants	N° haploid plants	N° diploid plants	N° aneuploid plants
A	Haploid	10	10	10	10	0	0

B	Aneuploid	40	12	3	0	0	3

C	Haploid	44	10	1	0	1	0

D	Haploid	2	2	2	2	0	0

**Figure 2:**
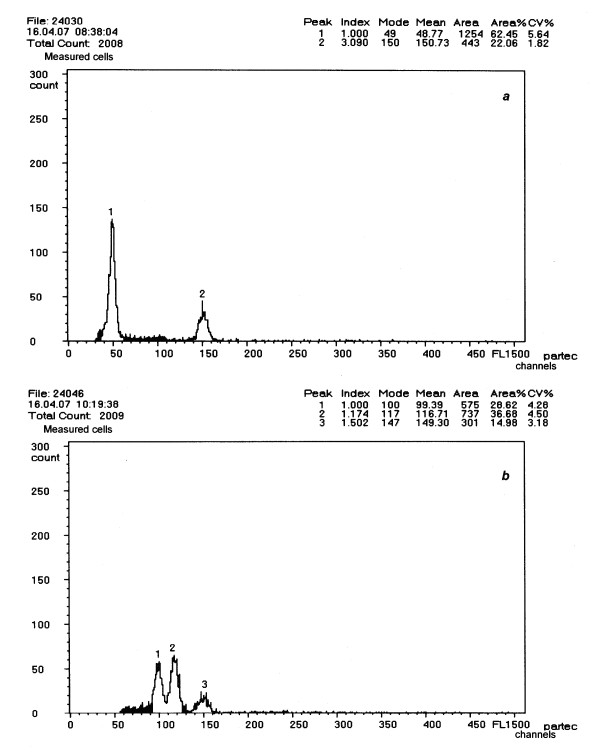
**Flow cytometry analysis**. *a*. Histogram of the G haploid plant (peak 1) and control triploid plant (peak 2). *b*. Histogram displaying a control diploid plant (peak 1), B.1 aneuploid plant (peak 2) and control triploid plant (peak 3).

Ploidy level was initially evaluated by flow cytometry. Eight of the nine plants obtained by direct germination of the embryos were haploid (Figure 2a) and one was diploid. The ploidy level of three of the four calli obtained (Table 1) was haploid, whereas one (callus B) was suspected to be aneuploid. The twelve plants obtained from haploid calli A and D were haploid. One diploid plant was obtained from haploid callus C, whereas we regenerated three plants with probable aneuploidy from callus B (Figure 2b). Seven haploid plants and the diploid plant from direct germination were very weak and died before making other characterizations.

Noteworthy, one of the propagations of the haploid plant G produced a branch with larger and wider leaves than those of the rest of the plant. The ploidy level of all leaves pertaining to this branch was determined by flow cytometry. Both diploid and haploid leaves were identified. All buds corresponding to the leaves that displayed diploid profiles were grafted in the greenhouse onto a vigorous rooststock. When buds sprouted and the leaves were completely formed, we again determined the ploidy level. Using this method, a diploid plant, arising from *in vivo *spontaneous somatic duplication of the chromosome number of the haploid line G was obtained and confirmed by chromosome counts.

**Figure 3:**
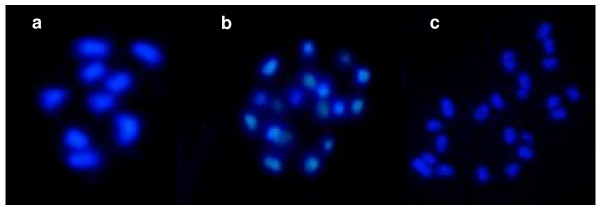
**DAPI stained chromosomes at the metaphase stage**. *a*. G haploid plant (2*n *= *x *= 9). *b*. C.1 double haploid plant (2*n *= 2*x *= 18). *c*. B.1 aneuploid plant (2*n *= 2*x*+4 = 22).

Chromosome counts were done on three lines and confirmed that haploid plant G had nine chromosomes (2*n *= *x *= 9) (Figure 3a), the diploid plant obtained from callus C had eighteen chromosomes (2*n *= 2*x *= 18) (Figure 3b) and we confirmed that the plants arising from callus B were aneuploid with twenty-two chromosomes (2*n *= 2*x *= 22) (Figure 3c).

### SSR analysis of plants obtained

**Figure 4:**
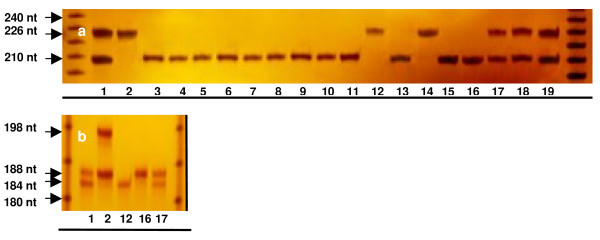
***a*. Ci03C08 SSR marker genetic analysis**. *b*. TAA 15 SSR marker genetic analysis. 1. 'Clemenules', 2. 'Fortune', 3. Haploid H, 4 - 11 Haploids obtained from embryogenic callus A, 12. Haploid G, 13. Haploid D.1, 14. Haploid E, 15. Haploid F, 16. Double haploid C.1, 17 - 19. Aneuploid plants obtained from embryogenic callus B.

All haploid, diploid and aneuploid plants established in the greenhouse, together with diploid 'Clemenules' clementine and 'Fortune' mandarin (the genotype used for irradiated pollen), were analysed with five SSR markers, heterozygotic in clementine. For each locus, all the haploid plants and the diploid plant C.1 possessed a single allele. All plants recovered from a same callus were identical for all markers. A restitution of clementine heterozygosity was observed only in the aneuploid plants for the markers Ci03C08, Mest 15 and TAA 15 (Figure 4).

Later, the haploid plant G, the diploid plant C.1 and the aneuploid plant B.1 were analysed with an additional 47 SSR markers to confirm their genetic structure (Table 2). 'Fortune'mandarin is a hybrid of clementine and 'Dancy' mandarin (*C. tangerina *Hort. ex Tan.). It is therefore impossible to find markers that fully differentiate 'Fortune' mandarin and 'Clemenules'clementine. However, of the 52 SSR markers analysed, 'Fortune' mandarin displayed 13 specific alleles in heterozygous status that were not present in clementine, or in any of the other three regenerated plants examined. The specific alleles of 'Fortune' mandarin are encountered in six linkage groups (Table 2) of the clementine genetic map [24].

**Table 2:** Genetic analysis of parentals and haploid, double haploid and aneuploid plants of 'Clemenules' with SSR markers.

Marker	Linkage group	Parental				Haploid		D. haploid		Aneuploid	
					
		Clemenules		Fortune		G		C.1		B.1	
						
		a_1_	a_2_	a_1_	a_2_	a_1_	a_2_	a_1_	a_2_	a_1_	a_2_
Ci02A09		161	162	161	162		162		162	161	162

Ci02B07		162	164	162	164	162		162		162	164

Ci02G12			250	**220**	250		250		250		250

Ci07A12		170		170	**178**	170		170		170	

Mest 056		155	165	155	165		165		165	155	165

Mest 088		134	138	134			138		138	134	

Mest 402		120	125	120	**150**	120			125	120	125

Mest 419		118	135		135	118			135	118	

Mest 431		260	270		270	260			270	260	270

Mest 458		215	225		225	215			225	215	225

Mest 473		216	224		224		224		224	216	224

Mest 488		136	146	146	**156**		146		146	136	146

Mest 506		164	168	164			168		168	164	

Mest 525		170	180	170	180	170			180		180

TAA 1		160	165	160	165		165		165	160	165

Ci02D09	1	229	237	229	237		237	229		229	237

Ci03C08	1	210	226		226		226	210		210	226

Ci05A05	1	146	154	146	**164**	146			154		154

Ci06B07	1	106	108	106	108		108	106		106	

CAC 15	1	150	159	150	159	150			159	150	159

TAA 41	1	148	154	**137**	148	148			154		154

Ci02C09	2	250	256	250	256		256		256	250	256

Ci02G02	2	113	123	113	123		123		123	113	

Mest 086	2	130	140		140		140	130			140

Mest 112	2	439	460	439			460	439		439	

Ci06B05	3	204	230	230	**236**	204			230	204	230

											

Mest 001	3	170	165	165		170		170		170	

Mest 246	3	248	256	248	256	248			256	248	

TAA 15	3	184	188	188	**198**	184			188	184	188

Mest 131	4	160	170	160		160		160			170

Mest 164	4	187	197	187	197		197	187		187	197

Mest 256	4	208	220		220		220	208		208	220

Mest 369	4	161	165	165	**189**		165	161		161	165

Mest 370	4	187	195	187	195	187		187		187	195

CAC 23	4	245	250	245	250	245			250	245	250

Ci02D04b	5	198	208	198	208		208	198		198	208

Ci03D12a	5	246	256		256	246			256	246	256

Ci07D06	5	155	175	155	175		175	155		155	175

Ci03B07	6	263	265	263	**276**		265	263		263	

Ci07C07	6	224	238	238		224			238		238

Mest 107	6	178	188	178		178		178		178	188

Ci02E08	7	260	275	260	275	260		260		260	275

Ci06A12	7	96	102	96	102	96			102	96	102

Ci07E12	7	120	126	120	126	120			126		126

Mest 015	7	184	188	184	188		188	184		184	188

Mest 154	7	105	108	105	108	105			108	105	108

Ci02F12	8	126	134	126		126		126		126	134

Mest 123	8	268	296	268	296	268		268		268	296

Mest 132	8	225	250	225	**230**	225		225		225	250

Ci07C09	9	246	260	246		248			260	246	

Ci08C05	9	151	173	**155**	173		173	151			173

Mest 291	9	178	190	178	**186**		190		190	178	190

Numbers indicate the size in nucleotids (nt) of the two alleles for each SSR marker.

Numbers in bold corresponds to specific alleles of 'Fortune'mandarin.

For all SSR markers analysed, the haploid plant G and the diploid plant C.1 possessed only one of the clementine alleles. Therefore, no restitution of maternal heterozygosity occurred for these markers for either the haploid or diploid plant (which, hereafter, is considered a double haploid). The aneuploid plant displayed incomplete restitution of maternal heterozygosity with a heterozygosity percentage of 61.5% concerning all linkage groups. With no specific allele of 'Fortune' mandarin, the aneuploid plants have a very low probability of being hybrids with this mandarin. Indeed taking into account only one marker of each of the six linkage groups with specific alleles the probability is (1/2)^6 ^(less than 0.016) The restitution of clementine heterozygosity for markers assigned to all linkage groups of the citrus genetic map indicate that the aneuploid plants could not have arisen from the spontaneous duplication of the chromosome stock of aneuploid callus cells with eleven chromosomes. Moreover, the incomplete restitution of the maternal heterozygosity discounts the hypothesis of somaclonal variation from maternal somatic tissue.

The diploid plant obtained from *in vivo *spontaneous somatic duplication of the chromosome number of the haploid line G was also confirmed fully homozygous for the same allele as the haploid plant G by using the same 52 SSR markers.

### Morphological characterization

**Figure 5:**
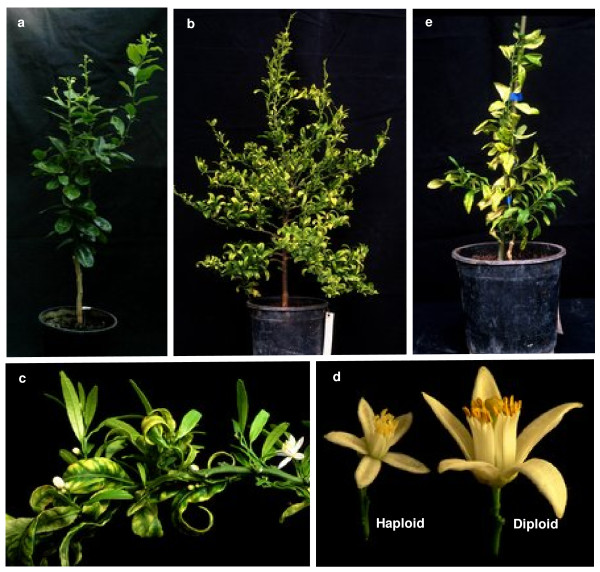
***a*. C.1 double haploid plant of 'Clemenules'**. *b*. G haploid plant of 'Clemenules'. *c*. Detail of blossom of the G haploid plant. *d*. Haploid and diploid flower of 'Clemenules'. *e*. B.1 aneuploid plant of 'Clemenules'.

There were statistically significant differences in all the variables analysed, according to the different ploidy level (Table 3 and Figure 5). The double haploid genotype had leaves with the greatest average foliar area (25.9 cm^2^), followed by the diploid 'Clemenules' clementine and the aneuploid genotype (19.4 and 4.0 cm^2^, respectively). The haploid plants had lower values, oscillating between 2.1 and 3.0 cm^2^, depending on the genotype.

**Table 3:** Measurements of leaves of haploid, diploid, double haploid and aneuploid plants of 'Clemenules'.

Genotype	Ploidy	Average leaf area (cm^2^)	Average leaf width (cm)	Average leaf length (cm)
Clemenules	Diploid	19.4^c^	3.3^e^	10.3^e^

B.1	Aneuploid	4.0^b^	1.4^c^	4.4^c^

C.1	Double haploid	25.9^d^	5.3^f^	7.2^d^

A.1	Haploid	2.1^a^	1.1^a^	3.2^b^

G	Haploid	3.0^ab^	1.1^a^	4.6^c^

D.1	Haploid	2.6^a^	1.2^ab^	3.8^b^

E	Haploid	2.9^a^	2.0^d^	2.3^a^

F	Haploid	2.3^a^	1.4^bc^	2.5^a^

Different letters in the same column indicate statistical differences at a significance level below 0.0001.

The double haploid plant also had the widest leaves (5.3 cm), followed by the diploid 'Clemenules' clementine and the haploid plant E (3.3 and 2.0 cm, respectively).

With respect to leaf length, the 'Clemenules' clementine had the largest value (10.3 cm), followed by the double haploid plant (7.2 cm). The haploid plants possessed a foliar length that varied between 2.3 and 4.6 cm. The maximum value of the haploid plants was similar to the leaf length of the aneuploid plant (4.4 cm).

### Histological characterization

**Figure 6:**
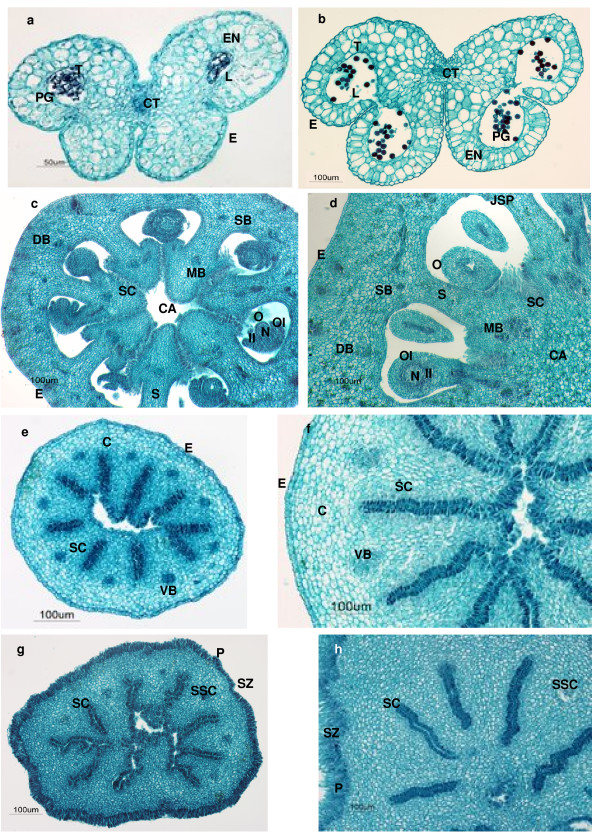
**Histological sections of haploid and diploid anthers, ovaries, styles and stigmas of 'Clemenules'**. *a*. Transversal section of haploid anther. *b*. Transversal section of diploid anther. *c*. Transversal section of haploid ovary. *d*. Transversal section of diploid ovary. *e*. Transversal section of haploid style. *f*. Transversal section of diploid style. *g*. Transversal section of haploid stigma. *h*. Transversal section of diploid stigma. C cortex, CA central axis, CT connective tissue, DB dorsal bundle, E epidermis, EN endothecium, II inner integument, JSP juice sac primordia, L locules, MB marginal bundle, N nucellus, O ovule, OI outer integument, P papilla, PG pollen grains, S septa, SB septal bundle, SC stylar canal, SSC stigmatic secretion canal, SZ stigmatic zone, T tapetum and VB vascular bundle.

The histological structure of anthers of the haploid plant G is similar to that of the diploid 'Clemenules' clementine (Figures 6a and 6b). Nevertheless, differences were observed in the width, height, percentage of anthers with locules and percentage of locules with pollen grains (Table 4). Values for width and height of the haploid anthers were, respectively, 58% and 64% of corresponding values for diploid plants. Diploid anthers always contained two locules with well-formed pollen grains, whereas only 4.7% of the haploid anthers possessed locules and the pollen grains were malformed.

**Table 4:** Measurements of histological sections of the anthers, ovaries, styles and stigmas of haploid and diploid plants of 'Clemenules'.

	Genotypes	
	
	Haploid G	Diploid 'Clemenules'
**Anthers**		
Width (μm)	324.9^a^	777.9^b^

Height (μm)	181.4^a^	504.1^b^

% anthers with developed locules	23.8^a^	100^b^

% anthers locules with pollen grains	4.7^a^	100^b^

**Ovaries**		
Diameter (μm)	702.2^a^	1428.7^b^

N° of locules per ovary	8^a^	10^b^

N° of ovules per section	6.6^a^	15.2^b^

**Styles**		
Diameter (μm)	440.6^a^	938.1^b^

**Stigmas**		
Diameter (μm)	591.1^a^	1496.4^b^

Different letters in the same line indicate statistical differences at a significance level below 0.0001.

In the ovaries of the haploid plant we observed a discontinuity in the central axis, which was not fused with the carpellous leaves (Figures 6c and 6d.). The diameter of haploid ovaries (Table 4) was approximately 50% smaller than those of diploid plants, although both displayed the same external morphology. Haploid ovaries had an average of eight locules per ovary, whereas the diploid plant contained ten locules. Haploid ovaries contained approximately half the number of ovules than diploid ovaries. At the same phenological stage, haploid ovules presented reduced growth compared to diploid plants in that the ovules of the diploid plant were totally developed whereas, for most of the haploid ovules, the inner and outer tegument did not completely surround the nucellus (Figures 6c and 6d).

The histological structures of haploid styles and stigmas were similar to diploid plants (Figures 6e, f, g and 6h). Haploid styles were 46 - 48% smaller than diploid styles and haploid stigmas were approximately 40% smaller than those of diploid plants (Table 4).

## Discussion

### Origin of the obtained plants

We have obtained haploid plants (2*n *= *x *= 9), aneuploid plants (2*n *= 2*x*+4 = 22) and a diploid plant (2*n *= 2*x *= 18) of 'Clemenules' clementine by *in vitro *culture of embryos produced following pollination with irradiated pollen of 'Fortune' mandarin.

The haploid plants originated from gamete reduction of 'Clemenules' clementine and regenerated by direct embryo germination or from embryogenic calli induced from the haploid seed embryos.

Genetic analysis with SSR markers confirmed the gynogenetic origin of all obtained plants, since we did not observe specific alleles of the male parent ('Fortune' mandarin) in any plant. No introgression of pollinator genome fragments, after gynogenesis induced by irradiated pollen, have been observed in citrus and other tree species [37,39,45]. However it should be noted that in Nicotiana [47,48] some introgression of male genome fragments have been identified in regenerated gynogenetic plant and confirmed by morphological markers analysis.

The diploid plant was obtained from an originally haploid callus, in which a spontaneous duplication of the chromosome stock took place. Double haploid [32,33] and trihaploid citrus plants [26] have previously been obtained by androgenesis. The duplication and triplication of the chromosome stocks of haploid clementine calli have been previously described [49]. Double haploid pummelos (*C. grandis *(L.) Osb) have also been obtained by colchicine treatment *in vivo *of axillary buds of haploid plants [50]. This plant is the first double haploid plant of 'Clemenules' clementine obtained by gynogenesis *in situ*, induced by irradiated pollen.

For the aneuploid plants, we propose that they arise from an aneuploid callus produced by an embryo derived, by gynogenesis, from a non-reduced aneuploid gamete (2*n *= 2*x*+4 = 22) of 'Clemenules' clementine. The formation of non-reduced gametes in citrus is a well-known phenomenon and is quite common in oranges and mandarins [51-54]. The abnormal meiosis that generates non-reduced gametes is also favourable for the formation of aneuploid gametes. Aneuploid citrus plants have previously been obtained in 2*x *× 2*x*, 2*x *× 4*x*, 4*x *× 2*x *and 2*x *× 3*x *hybridisations [43,55], but this is the first report of an aneuploid plant obtained by gynogenesis of a non-reduced aneuploid megagametophyte.

### Effect of ploidy level on the morphologic characteristics of regenerated plants

Significant differences in leaf morphology and size were observed in relation to ploidy level. A diversity of leaf morphologies was also observed among the haploid plants, obtained from independent meiotic events.

The double haploid plant of 'Clemenules' clementine displayed shorter internodal segments, thorns, a more robust appearance and more vigorous growth than the majority of haploid plants. Nevertheless with time, dieback of branches was observed and periodical propagations were needed to avoid the loss of this genotype. Duplication of the ploidy level (from haploid to double haploid) increased the area, length and width of leaves. The floral organs of haploid clementine were smaller than those of the diploid parental plant. The haploid ovaries, styles and stigmas were approximately 40 - 50% smaller than those of diploid plants. A comparative histological study of the foliar structure of diploids and autotetraploid plants of 'Valencia' sweet orange and 'Femminello' lemon, Romero-Aranda *et al*., [56] revealed that an increase in ploidy level was associated with an increase in cellular volume, leading to autotetraploid leaves between 20 - 30% thicker than in diploid plants. In plants of *Solanum phureja *with the same genetic structure but with different levels of ploidy (1*x*, 2*x *and 4*x*), a progressive increase in nuclei and cell volumes was coincident with increasing ploidy. In addition, 2*x *and 4*x *plants showed greater development and vigour than 1*x *plants [57]. Similarly, haploid *Vacccinum *spp. and apple tree plants also display a very weak appearance and poor growth compared with diploid plants [38,58].

Plant vigour seems to be particularly affected by haploidy in most citrus species. All haploid plants of clementine, obtained by androgenesis or gynogenesis, displayed a weak appearance and poor growth [25,31,40,44,45] and most plants typically died in the test tube or in the greenhouse. The weakness of haploid plants could be due to the expression of recessive deleterious or lethal genes [32,44]. The presence of such genes in a heterozygous state in parental genotypes could be enhanced in citrus by generalized vegetative propagation. Nevertheless, in our work, we obtained a haploid plant of clementine (Line G) manifesting much more vigorous growth than the other haploid clementine plants, and it flowered after four years. This plant has been propagated in a greenhouse on different rootstocks of citrus and its culture does not require special care. It has also been grafted in the field, where it is cultivated under the same conditions as the commercial citrus varieties.

The absence of locules has been observed very frequently in the anthers of haploid plants and the rare locules always contained abnormal pollen grains. Pummelo haploid flowers also contained fewer pollen grains than those of diploid plants and displayed very low viability [58] and similar observations have also been made in *Prunus *[59].

The ovaries of some of the haploid plants characterized here were abnormal, having carpellous leaves not fused with the central axis. This anatomic characteristic may prevent the pollen tube from reaching the ovule and, thus, contribute to the lower number of ovules per ovary in the haploid plant compared to the diploid plant. Thus, in addition to the abnormal meiosis associated with haploidy, the anatomical characteristics of haploid plants can explain unsuccessful hybridisations, so far, using the pollen of different citrus genotypes. Yahata et al [58] suggested that the use of haploid plants for improvement programs or genetic research was very complicated.

### Use of the haploid clementine line G for further genetic studies

The development of a double haploid line of the G haploid plant was considered an important objective, within the perspective of potential use in further genetic studies. Spontaneously a double haploid plant was obtained from the haploid plant G by somatic duplication of chromosome number. Duplication of the chromosome stock of nucellar cell-producing embryos is frequent in apomictic diploid plants of citrus [60]. However, it is rare in somatic vegetative tissue of diploid plants [61] although Yamamoto and Tominaga [62] obtained a haploid-diploid periclinal chimera from a haploid plant recovered in a cross between clementine and a triploid hybrid. This double haploid line is still young and, therefore has not been characterized yet from the reproductive point of view. Haploid and double haploid plants, together with the original diploid plant and autotetraploid plants obtained in our laboratory [63] are excellent material to carry out pioneering experiments in citrus in terms of examining the genetic and epigenetic changes that can take place as a result of genic dose and for further genetic studies in relation to the whole citrus genome sequencing project.

## Conclusion

Haploid plants are of great interest for structural genomics. The ICGC decided to select a haploid plant to establish a reference whole citrus genome sequence. Indeed, such a plant would significantly facilitate the assembly of sequences given the high level of heterozygosity of most citrus species. In our study, gynogenesis *in situ *induced by irradiated pollen, has allowed to obtain haploid, double haploid and aneuploid plants of 'Clemenules' clementine. Haploid plants were obtained by direct germination of embryos without a callus phase or from haploid embryogenic calli induced from haploid seed embryos. The haploid plants displayed significant morphological diversity, but most of them grew weakly. Only haploid plant G, obtained directly from embryo germination without a callus phase, displayed vigorous growth and was the only haploid clementine plant to produce flowers. Furthermore, a double haploid plant derived from this line was also obtained by spontaneous somatic doubling of the chromosome stock of a vegetative bud.

This haploid plant, along with one haploid plant obtained in France [25] and one trihaploid plant from Italy [26], have been analyzed exhaustively in different laboratories around the world with a high number of SSR markers and microarray platforms. The ICGC has chosen the haploid plant G, developed and presented here, to establish the complete reference sequence of the nuclear genome of citrus ant it is now being used for this purpose.

## Methods

### Plant material

'Clemenules' clementine was used as female parent and 'Fortune'mandarin was selected as male parent due to its high pollen fertility and compatibility with the clementine group. Flowers of 'Fortune' mandarin was collected in preanthesis and were irradiated with gamma rays with a single dose of 500 Gy from a cobalt 60 source.

### Pollination and embryo rescue

Pollinations were carried out in trees growing in a greenhouse. Three-hundred and fifty flowers of 'Clemenules' clementine were pollinated manually by placing an irradiated anther in the stigma of each flower. Fruits were collected at maturity and seeds were extracted and surface sterilized with a sodium hipochloride solution (0.5% active chlorine).

Embryos were isolated from underdeveloped seeds in aseptic conditions with the aid of a stereoscopic microscope and cultivated on Petri dishes containing the Murashige and Skoog [64] culture media with 50 g/L sucrose, 500 mg/L malt extract supplemented with vitamins (100 mg/L i-inositol, 1 mg/L pyridoxine hydrochloride, 1 mg/L nicotinic acid, 0.2 mg/L thiamine hydrochloride, 4 mg/L glycine) and 8 g/L Bacto agar (culture media MS). After germination plants were transferred to 25 × 150 mm test tubes with MS culture media without malt extract.

Embryogenic calli were cultivated in MS culture media with 40 g/L of sucrose and 1.8 g/L gelrite. Embryos obtained from these calli were cultured in MS culture media with 0.02 mg/L α-naftalenacatic acid and 1 mg/L gibberellic acid to promote germination. Cultures were maintained at 24 ± 1°C, 60% humidity and 16 h daily exposure to 40 μE m^-2 ^s^-1 ^illumination.

### Plant regeneration

The germinated embryos did not develop roots or, at most, very small and weak ones. For this reason *in vitro *shoot tip grafting was used for plant regeneration [46]. Micrografted plants were cultivated in a liquid nutrient culture medium composed of the plant cell salt solution of Murashige and Skoog [64] with vitamins (100 mg/L i-inositol, 1 mg/L pyridoxine hydrochloride, 1 mg/L nicotinic acid and 0.2 mg/L thiamine hydrochloride) and 75 g/L sucrose. The medium was distributed into 25 × 150 mm test tubes in 25 mL aliquots. A folded paper platform, perforated at its centre for insertion of the root portion of the rootstock, was placed in the nutrient solution. The cultures were kept at a constant 24 ± 1°C and exposed 16 h daily to 40 μE m^-2 ^s^-1 ^illumination.

### Ploidy level analysis

Ploidy level was determined by flow cytometry. Each sample consisted of a small leaf piece (~0.5 mm^2^) collected from each plant with a similar leaf piece from a diploid or triploid control plant. For embryogenic calli, two sample types were taken: one sample was just composed of a fragment of the callus, and the other from a fragment of callus with a piece of leaf from a control plant. Samples were chopped together using a razor blade in the presence of a nuclei isolation solution (High Resolution DNA Kit Type P, solution A; Partec, Münster, Germany). Nuclei were filtered through a 30-μm nylon filter and stained with a DAPI (4-6-diamine-2-phenylindol) (High Resolution DNA Kit Type P, solution B; Partec) solution. Following a 5 min incubation, stained samples were run in a Ploidy Analyzer (Partec, PA) flow cytometer equipped with a HBO 100-W high-pressure mercury bulb and both KG1 and BG38 filter sets. Histograms were analyzed using the dpac v2.0 software (Partec), which determines peak position, coefficient of variation (CV), and the relative ploidy index of the samples.

### Genetic analysis

Genetic analysis was carried out with SSRs markers. The extraction of genomic DNA was done according to Dellaporta and Hicks [65] with small modifications. The plants obtained were analysed with heterozygotic markers for clementine [19,66,67]. These markers are positioned broadly throughout the genetic map of clementine [24].

PCR product conditions and separation were by means of vertical denaturalized electrophoresis (bis-acrylamide acrylamide 6%, urea 7 M) buffer TBE 0.5× (Tris, boric acid and EDTA 0.5 M, pH 8) in a DCodeTM Biorad, according to the methodology described by Froelicher *et al*. [45]. The amplified fragments were detected by means of silver staining [68].

### Chromosome counts

Chromosome counts were determined according to the methodology described by D'Hont *et al *[69] and observations were carried out with ultraviolet light in an E800 eclipse Nikon microscope.

### Morphological characterization

Thirty adult leaves located in the intermediate zone of spring shoots were taken from each plant. Area, width and foliar length were measured with LiCor 3100 C equipment.

### Histological characterization

Twenty flowers of diploid 'Clemenules' clementine and the haploid plant G, were collected in preanthesis. Ovaries, styles and stigmas were fixed in FAA (formaldehyde, glacial acetic acid and alcohol 50%). The samples were embedded in paraffin, cut in portions of 10 μm and dyed with safranin and fast green according to the general methodology described by Jensen [70]. Twenty sections of the intermediate zone of each organ were analysed. Observations were made with an E800 Eclipse Nikon microscope.

### Statistical analysis

A hierarchized ANOVA was carried out for each of the measured characters, applying the transformation square root (v) for the variable area (cm^2^). The model used was: x_ijk _= μ + P_i _+ (G)_j(i) _+ ω k(ij). Later the adjusted measurements were compared using Bonferroni correction.

The normality of the variables was verified with the Kolmogorov test and an ANOVA was made of the all variables analysed between anthers, ovaries, styles and stigmas. The model used was: x_ij _= μ + G_j _+ ω_ij_.

## Authors' contributions

PA carried out the pollination, culture tissue, flow cytometry, morphological characterization, chromosome counts, genetic analysis and wrote the manuscript, JJ carried out pollination, culture tissue, flow cytometry and chromosome count, MH carried out flow cytometry and DNA extractions, JP cultured plants in the greenhouse, PO carried out genetic analysis, writing, organization and discussion of the manuscript and LN conceived of the study, and participated in its design and coordination, and revised the manuscript. All authors read and approved the final manuscript.
